# Mitochondrial Inverted Repeats Strongly Correlate with Lifespan: mtDNA Inversions and Aging

**DOI:** 10.1371/journal.pone.0073318

**Published:** 2013-09-17

**Authors:** Jiang-Nan Yang, Andrei Seluanov, Vera Gorbunova

**Affiliations:** Department of Biology, University of Rochester, Rochester, New York, United States of America; University of Medicine and Dentistry of New Jersey, United States of America

## Abstract

Mitochondrial defects are implicated in aging and in a multitude of age-related diseases, such as cancer, heart failure, Parkinson’s disease, and Huntington’s disease. However, it is still unclear how mitochondrial defects arise under normal physiological conditions. Mitochondrial DNA (mtDNA) deletions caused by direct repeats (DRs) are implicated in the formation of mitochondrial defects, however, mitochondrial DRs show relatively weak (Pearson’s *r* = −0.22, *p*<0.002; Spearman’s *ρ* = −0.12, *p = *0.1) correlation with maximum lifespan (MLS). Here we report a stronger correlation (Pearson’s *r* = −0.55, *p*<10^–16^; Spearman’s *ρ* = −0.52, *p*<10^–14^) between mitochondrial inverted repeats (IRs) and lifespan across 202 species of mammals. We show that, in wild type mice under normal conditions, IRs cause inversions, which arise by replication-dependent mechanism. The inversions accumulate with age in the brain and heart. Our data suggest that IR-mediated inversions are more mutagenic than DR-mediated deletions in mtDNA, and impose stronger constraint on lifespan. Our study identifies IR-induced mitochondrial genome instability during mtDNA replication as a potential cause for mitochondrial defects.

## Introduction

Mitochondria are responsible for generating most of ATP in a eukaryotic cell. Mitochondria have their own genome, which is a small DNA molecule between 13 kb and 26 kb in most animals, encoding central components of the electron transport chain and some rRNAs and tRNAs. Mitochondria play the central role in the signal transduction of apoptosis and have been linked to cell loss and aging [1], [2].

The role of mitochondria in aging was proposed 40 years ago [3], and is still a subject of a hot debate [1], [4]–[12]. Mitochondrial free radical theory of aging proposes that reactive oxygen species that are produced in the mitochondria, cause damage to macromolecules such as proteins, lipids and mtDNA and organisms age as they accumulate free radical damage over time over time [3]. This theory is supported by the findings that the catalase targeted to mitochondria attenuates aging in mice [13]. However, accumulating evidence argues against a simple link between free radical damage and aging [1], [5]–[7].

The role of mtDNA in aging was first observed in filamentous fungus *Podospora anserina* [14]–[16]. Later, mtDNA point mutations and deletions have been found to accumulate in aging animal tissues [4], [17]–[20]. MtDNA deletions accumulate to high levels in post-mitotic tissues, such as brain, heart, and skeletal muscle, and the fraction of deletions could exceed 60% in neurons [21], [22]. Deletions were also suggested to be a driving force behind premature aging in mitochondrial mutator mice [23]. Deletions often arise between direct DNA repeats [24], [25], and the frequency of direct repeats (DRs) in mtDNA was shown to negatively correlate with mammalian lifespan [26], [27], suggesting that more DRs are associated with more deletions and faster aging.

Here we report that mitochondrial inverted repeats (IRs) have a stronger negative correlation with animal lifespan than DRs, and that IRs induce mitochondrial genome instability during mtDNA replication and cause mtDNA inversions that accumulate with age in mice.

## Results and Discussion

### Inverted Repeats (IRs) have a Stronger Correlation with Maximum Lifespans (MLS) than DRs

**Figure 1:**
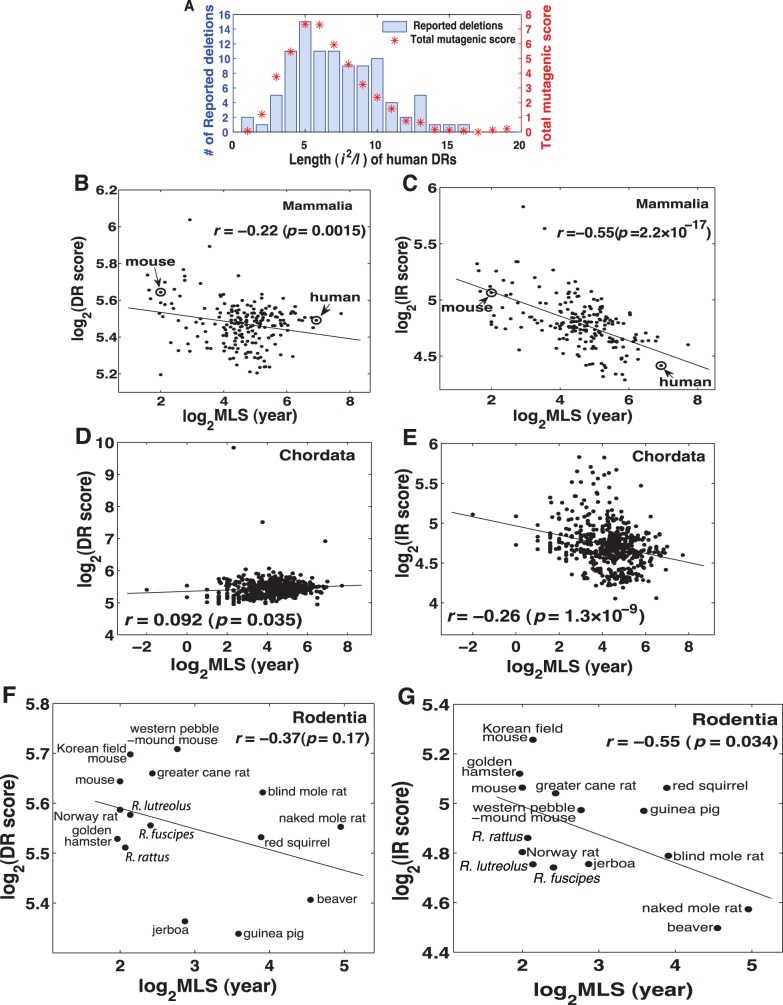
Inverted repeats (IRs) show stronger negative correlation with maximum lifespan (MLS) than direct repeats (DRs). (**A**) The mutagenic score calculated as 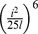 where *i* is the number of identical matches in *l* bps for each DR length, has a good fit with the number of experimentally reported human mtDNA deletions. The human mtDNA deletions are from MITOMAP database [30]. (**B, C**) The correlation between total mutagenic score, calculated as a sum of mutagenic scores of all DR (**B**) and IR (**C**) lengths for each species, and MLS within mammals. (**D, E**) The correlation between total mutagenic score and MLS within Chordata. (**F, G**) The correlation within Rodentia. *R.*, *Rattus*.

We analyzed the correlation between mtDNA IRs and lifespans for all animal species (a total of 529) that had both mitochondrial genome reference sequences in NCBI and lifespan information in AnAge database [28] as of March 1, 2012. We used our algorithm, RollingRepeat, to accurately count repeats of all lengths. For any repeat between two genomic positions, RollingRepeat extends it to the longest matched sequence. To quantify the burden of repeats of different lengths, we calculated a mutagenic score for each repeat with *i* identical matches in *l* bps according to a formula 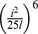, and then added all repeats (excluding D-loop that is often highly repetitive and not well sequenced) up to obtain a single score for each species. The formula was derived from the empirical relationship between repeat length and DR-mediated deletion rate in yeast mitochondria [29]. We then tested the formula with MITOMAP database [30], which contains reported human mitochondrial deletions. The total mutagenic score of all DRs of each length matched well with the reported number of mtDNA deletions in human (Fig. 1A).

The mutagenic score of IRs had a stronger Pearson’s correlation with MLS than DRs (Fig. 1B, 1C) in all the available 202 mammalian species, with *r = *–0.55 for IRs and *r = *–0.22 for DRs. The negative correlation did not exist for DRs in all Chordata (Fig. 1D) but existed for IRs in Chordata (*r* = –0.26, Fig. 1E) (and in Animalia, *r* = –0.31, Fig. S1) even after excluding Mammalia (*r* = –0.22, *p*<10^–4^), suggesting that IR-mediated mutagenesis is a universal mechanism involved in animal aging. Since our data did not meet normality assumption, we also calculated Spearman’s *ρ*, which only slightly affected the correlations for IRs but removed the significant negative correlations for DRs (Table 1). The weaker correlation between IRs and MLS outside Mammalia may be due to the diverse physiology of these animals. We took a closer look at the order Rodentia that contains several important model animals and has a wide range of MLS. Long-lived rodents, such as naked mole rat and beaver, have fewer IRs but not DRs than short-lived rodents (Fig. 1F, 1G). All the above negative correlations between IRs and MLS remained significant after phylogenetic correction by phylogenetically independent contrasts [31] (Table 1). To confirm the robustness of our RollingRepeat algorithm for repeat discovery, we used NCBI blastn program to identity repeats and the results were similar (Fig. S2).

**Table 1:** Correlations of DRs and IRs with MLS in different clades.

Clades	Species #	DRs		IRs	
		Pearson	CAIC	Pearson	CAIC
Animalia	529	−0.03	−0.07	−0.31*	−0.23*
Chordata	524	0.09	−0.05	−0.26*	−0.21*
Animalia – Mammalia	327	−0.02	−0.07	−0.29*	−0.19*
Chordata – Mammalia	322	**0.14**	−0.04	−0.22*	−**0.17**
Mammalia	202	−**0.22**	−0.13	−0.55*	−0.34*
Carnivora	40	−0.26	−**0.33**	−**0.47**	−**0.46**
Primates	36	0.07	−0.18	−**0.47**	−0.25
Artiodactyla	34	−**0.35**	−0.24	−0.57*	−**0.51**
Cetacea	22	0.23	0.09	−0.16	−0.10
Rodentia	15	−0.37	−0.13	−**0.55**	−**0.57**
Diprotodontia	9	0.33	0.21	−0.35	−0.48

**Bold** font, *p*<0.05; *, *p*<0.001. Two-tailed. CAIC, comparative analysis by phylogenetically independent contrasts. –Mammalia, excluding Mammalia. NCBI taxonomic information was used to construct the phylogenetic tree and assuming equal branch lengths. The correlations of IRs and MLS decreased from –0.55 to –0.34 for mammals but were still significant (*p* = 7.2×10^−7^). So, the strong correlation between IRs and MLS is not a false correlation caused by phylogeny.

### Short Repeats also show Negative Correlation with MLS

**Figure 2:**
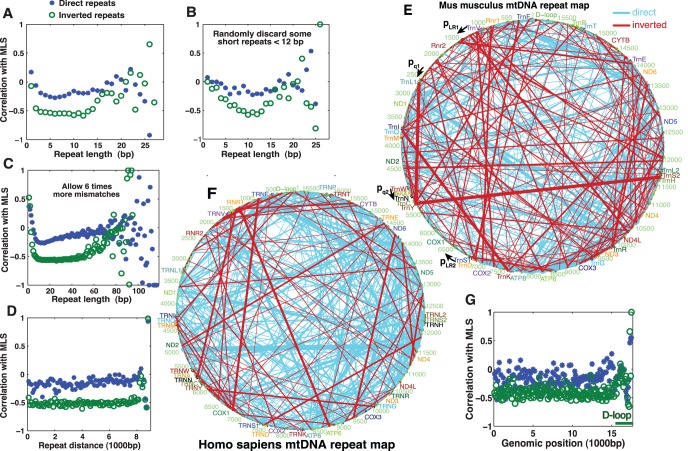
The influence of repeat length, distance between repeats, and genomic position on the correlation between mutagenic score and maximum lifespan (MLS). (**A**) Effect of repeat length on the correlation between mutagenic score and MLS. (**B**) Short repeats show weaker correlation with MLS if their total number is made equal to the number of long repeats by randomly discarding some of the short repeats. Each repeat was discarded with probability 1–3*^i^* ^–12^ (*i* is the number of identical matches) so that repeats shorter than 12 bps had nearly the same expected number (3 is used instead of 4 because 1/4 shorter repeats, which were inside longer repeats, were not counted). (**C**) Effect of mismatches on the correlation between mutagenic score and MLS. (**D**) Effect of the distance between repeats on the correlation between mutagenic score and MLS. (**E, F**) Repeat maps showing all repeats longer than 10 bp. Line thickness is proportional to repeat length, with the thinnest lines representing the shortest repeats (11 bps), and the thickest lines representing repeats of about 19 bp. p_LR1_ and p_LR2_ indicate the positions of PCR primers used for long range (LR) PCR; p_q1_ and p_q2_ indicate the positions of primers used for qPCR. (**G**) Effect of genomic position of the repeats on the correlation with MLS. The starting position of tRNA-Phe is used as position 1 for all mammals. The correlations at the end of D-loop were not statistically significant, as sequence information for the D-loop is only available for a small number of species.

IRs have a strong negative correlation with mammalian MLS even for repeats as short as 2 bps (*r* = –0.46, *p*<10^–11^; Spearman’s *ρ* = −0.47, *p*<10^–11^, Fig. 2A). Short repeats may be important in aggregate, because the total repeat number increases exponentially with the decrease of repeat length. Short DRs also showed a significant, albeit weaker, negative correlation (*p*<0.03 if *r*<–0.15, two-tailed) with MLS. This is contrary to previous studies that did not use an algorithm specifically for short repeat discovery and found no correlation between DRs and MLS for repeats shorter than 10 bps [26], [27]. The correlation for a long repeat length was calculated after excluding species that do not have repeats of that length. The weaker correlation for the longest repeats was likely due to the small total number of long repeats. Similarly, after randomly discarding short repeats so that their expected numbers were close to the 12-bp repeats of the same species, the correlations for short repeats also decreased (Fig. 2B).

### Correlation of IRs and DRs with MLS Tolerates Mismatches and Variations in Spacer Length

By default, we allowed a mismatch every three matches. However, when allowing two mismatches every match, even very long repeats had a strong negative correlation with MLS (Fig. 2C), suggesting that repeats with multiple mismatches still exert an effect on aging. The length of the spacer sequence between the two repeats had a negligible effect on the correlation, especially for IRs (Fig. 2D), suggesting that repeats are capable of long-distance interaction.

### Repeats Correlate with MLS throughout the Mitochondrial Genome in Mammals

Repeats are found throughout the mitochondrial genome. Figures 2E, 2F show repeat maps of all repeats longer than 10 bps for mouse and human. Repeat maps for yeast *S. cerevisiae* and the 529 animal species analyzed are available upon request. Arrangement of genes in mtDNA is conserved in mammals. The strong correlation between repeats and MLS exists throughout the mitochondrial genome except for the D-loop, which showed weaker correlations with MLS (Fig. 2G).

### IRs cause Inversions in Mouse mtDNA

**Figure 3:**
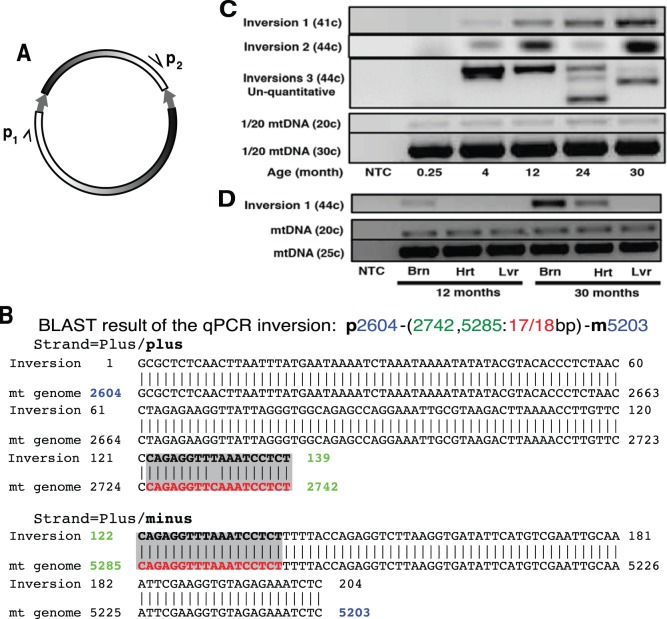
IRs-mediated inversions accumulate with age in mouse brain and heart. (**A**) Primer pair p_1_ and p_2_ amplifying inversion will not generate a PCR product on a normal mtDNA template. (**B**) Example of an inversion (inversion #1) obtained by sequencing a PCR product. The IR sequence that mediated the inversion is shaded gray. The plus strand of the inversion matches a sequence on the plus strand and a sequence on the minus strand of the mitochondrial (mt) genome. (**C**) Inversions amplified from the mouse brain. Different inversions were amplified by different primer pairs (Materials and Methods 5). The number of PCR cycles is indicated in parentheses. (**D**) Inversions accumulate in brain and heart. NTC, no template control.

IRs can cause inversions in bacterial and eukaryotic chromosomes [32]–[34]. To test whether inversions are generated in mtDNA, we designed PCR primers that anneal to the same DNA strand, with one primer being outside of the repeat pointing towards the repeat, and the second primer inside the spacer region. These primers can yield a product only after an inversion occurs in mtDNA (Fig. 3A). We used seven such primer pairs amplifying inversions caused by different IRs in C57BL/6 mouse brain and heart. Sequencing confirmed that the PCR products corresponded to inversions in the mtDNA (Fig. 3B and Table 2).

**Table 2:** Mouse mtDNA inversions and interrupted genes.

Inversions*	Genes interrupted
**p**2532-(2742,5285∶17/18 bp)-**m**4770	TrnL1 TrnY
**m**2903-(2725,5268∶17/18 bp)-**p**5499	TrnL1 TrnY
**p**3448-(3721,5964∶8/9 bp)-**m**4770	TrnI COX1
**p**3448-(3689,5997∶8/8 bp)-**m**4770	ND1 COX1
**p**3448-(3903,5281∶12/12 bp)-**m**4770	TrnM TrnY
**p**3448-(3906,5114∶16/19 bp)-**m**4770	TrnM TrnN
**p**3448-(3483,4940∶0/0 bp)-**m**4770	ND1 ND2
**p**3448-(3519,7592∶2/3 bp)-**m**7434	ND1 COX2
**p**3448-(3706,7469∶5/5 bp)-**m**7434	ND1 COX2
**p**3448-(3506,7664∶1/1 bp)-**m**7434	ND1 COX2
**p**3448-(3568,7598∶2/2 bp)-**m**7434	ND1 COX2
**p**3448-(3646,7596∶0/0 bp)-**m**7434	ND1 COX2
**p**3448-(3551,7679∶4/4 bp)-**m**7434	ND1 COX2
**p**3448-(3506,7750∶0/0 bp)-**m**7434	ND1 TrnK
**p**3561-(3787,7650∶18/20 bp)-**m**7520	TrnQ COX2
**p**8392-(8521,6876∶17/19 bp)-**m**6791	TrnS1 ATP6
**p**11800-(11931,10531∶19/22 bp)-**m**10401	ND4 ND5
**p**2590-(5287,2742∶18/20 bp)-**m**2590	TrnL1 TrnY
**p**2590-(2668,3384∶3/4 bp)-**m**2590	16SrRNA ND1
**m**5405-(4136,3417∶16/18 bp)-**p**5405	ND1 ND2

* Format: **m**/**p**p_1_-(j_1_,j_2_:*i/l* bp)-**m**/**p**p_2_. **m**, **m**inus strand; **p**, **p**lus strand. p_1_, p_2_, the starting positions of the two primers. j_1_, j_2_, positions of the last nucleotides of the two strands at the inversion junction: *i* identical matches in *l* bps. All positions were from BLAST results as in Fig. 3b. The last three inversions were amplified by single primers.

### Inversions Accumulate with Age in Mouse Brain and Heart

We next tested whether the frequency of inversions increases with mouse age. Three sets of primers amplifying across different repeats were used in a semi-quantitative PCR reaction normalized to un-rearranged mtDNA. No inversions were detected in the brains of one-week old mice. Inversions appeared in 4 months old mice (Fig. 3C). The frequency of the inversion #1 increased with age. Inversions #2 and #3 showed a more complex behavior suggestive of secondary rearrangements taking place in older animals (Fig. 3C). We next tested for inversion #1 in the brain, heart, and liver of 12- and 30-months old mice (Fig. 3D). No inversions were detected in the liver in both ages, and in the 12-months old heart, while brain and heart of 30-months old animals showed inversions. These results suggest that inversions tend to accumulate with age in tissues with high-energy metabolism.

PCR amplification can potentially give rise to artifacts such as deletions or inversions. The following experiments argue that the inversions we observed in aged brain and heart were not a result of a PCR artifact. First, PCR amplification using the same sets of primers and DNA from the livers of 20 young and 20 old mice did not show inversions. Second, inversions in brain and heart displayed age-related pattern. Third, the same sets of PCR primers did not detect any inversions in the DNA from a mouse fibroblast cell line even after 50 PCR cycles. In the latter experiment an equal amount of mtDNA from a 24-month-old mouse brain was used as a positive control that showed inversions before 40 cycles on the same PCR plate. Interestingly, previous analysis [35] using high-throughput sequencing identified a large number of inversions, although the inversions were deemed to be sequencing errors.

**Figure 4:**
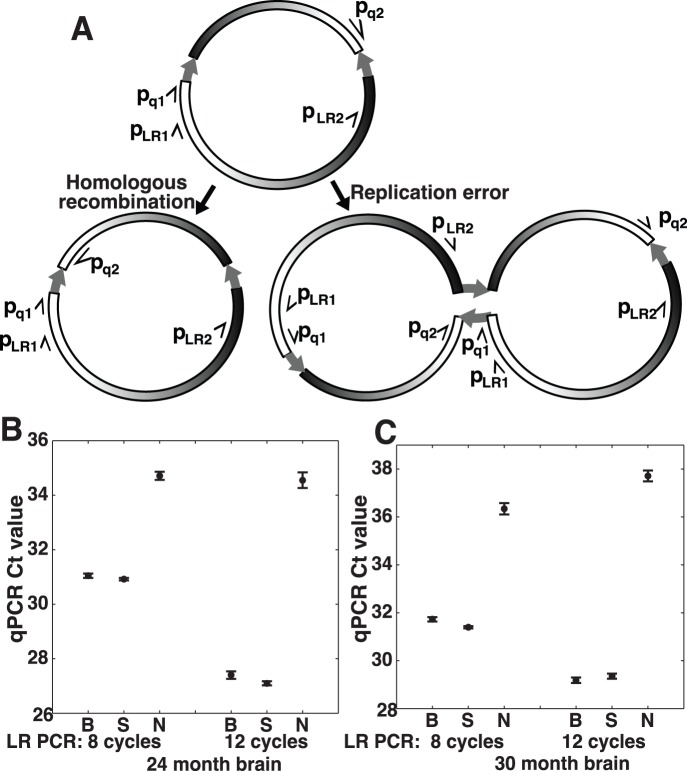
The majority of inversions are caused by mtDNA replication. (**A**) Diagram showing inversion products generated by recombination or replication mechanisms. IRs are indicated by thick arrows. Thin half-arrows indicate LR PCR primers (p_LR1_ and p_LR2_) and qPCR primers (p_q1_ and p_q2_) used to quantify the inversion. (**B**) Quantification of the recombination and replication inversion products using qPCR. Total DNA from a 24-months-old mouse brain was first amplified with 8 or 12 cycles of LR PCR with the primers p_LR1_ and p_LR2_ (B), p_LR1_ alone (S), or no primer control (N). Inversions resulting from replication errors can be amplified with p_LR1_ alone, while inversions resulting from homologous recombination require both p_LR1_ and p_LR2_ primers._._ The LR PCR was followed by qPCR with primers p_q1_ and p_q2_ to specifically quantify the inversion. The Ct values, inversely proportional to log of template concentration, are plotted for each PCR reaction. Error bars indicate s.e.m. (*n = *6 for N; *n* = 12 for groups B and S). (**C**) A replicate of (**B**) using the total DNA from a brain of a different mouse (30-months-old).

Quantitative PCR (qPCR) revealed that the relative concentration of the inversion #1 in Fig. 3B in 30 months old mouse brain was about 1 in 62900 mtDNAs. Since the corresponding IR had 17 matches in 18 bps and a mutagenic score of (17^2^/25/18)^6^ = 0.0702, and the whole mouse mitochondrial genome has a total IR mutagenic score of 36.3 (32.6 excluding D-loop), this gives an estimate of 1 inversion in every 62900×0.0702/36.3 = 122 mtDNAs. This number seems too low to drive aging. However, given that inversions can induce complex secondary rearrangements (Fig. 3C; Fig. 4) which would not be detected by the original set of primers the actual frequency of inversions may be much higher.

### IR-mediated Inversions are Generated through mtDNA Replication

Two models were proposed to explain IR-mediated inversions [36] (Fig. 4A). In the first model, inversions are generated by homologous recombination repair of damaged DNA, resulting in a simple inversion of the sequence between the repeats. In the second model, inversions are generated via DNA replication error, producing a head-to-head dimeric circular DNA molecule. Remarkably, duplicated mtDNA molecules were shown to accumulate in aged human tissues [37]. A simple inversion can be distinguished from a mtDNA dimer using long range (LR) PCR with a single primer amplifying towards the repeat (Fig. 4A). Such PCR reaction will only yield a product on a head-to-head dimeric template. The existence of such dimers was confirmed by sequencing the inversions generated with a single primer (the last three products in Table 2).

To compare the levels of head-to-head dimers and simple inversion products we performed an 8- or 12-cycles of LR PCR nested with a qPCR. The LR PCR reactions contained either two primers, p_LR1_ and p_LR2_, a single primer p_LR1_ (Fig. 2E & Fig. 4A), or no primers. Reactions containing two primers are able to amplify both the simple inversions and head-to-head dimers caused by the same IR, while a single primer can only amplify the head-to-head dimer. Following LR PCR, a qPCR using primers p_q1_ and p_q2_ (Fig. 2E & Fig. 4A) was used to specifically quantify the inversion amplified in the LR PCR. The qPCR Ct value (inversely proportional to log of template concentration) after the single-primer LR PCR was nearly the same with the Ct value after the two-primer LR PCR (Fig. 4B). The calculated ratio of template concentrations of single-primer-amplifiable inversions to two-primer-amplifiable inversions ranged from 92% to 119%, and is larger than 66% with 95% confidence (*n = *12) for the lowest replicate (12 LR cycles for 30 month brain in Fig. 4C), suggesting that the majority of, if not all, inversions are generated through replication-dependent mechanism. This is consistent with the hypothesis proposed based on the studies of mutator mice that mtDNA damage arises as a result of errors by mtDNA polymerase [25].

### Why do IRs have a Stronger Correlation with MLS than DRs?

The dimeric mtDNA molecule caused by IRs is itself a large inverted repeat (Fig. 4A), which is highly unstable and can cause additional complex rearrangements. This may explain why some of the inversions did not show a perfect gradual increase with age in Fig. 3C and why some inversions and deletions did not have a repeat at the junction (Table 2). Another explanation is that inversions and deletions may have a dominant-negative effect. mtDNA deletions and inversions can interrupt mitochondrial genes or create hybrid gene products by generating novel junctions. These hybrid proteins and RNAs may disrupt proteostasis and impair mitochondrial function. Since nearly all mtDNA consists of coding sequences, IRs may be more harmful than DRs because, an inversion produces two aberrant products, while a deletion produces only one.

In summary, the correlation we uncovered between the IRs in the mtDNA and lifespan highlights mtDNA inversion as a type of mtDNA rearrangement having a strong connection to lifespan. We propose a model where IRs and DRs in the mitochondrial genome cause mtDNA inversions and deletions during mtDNA replication, these mtDNA rearrangements accumulate with age, disrupt ATP production, trigger apoptosis, and promote aging and age-related diseases.

## Materials and Methods

### Ethics Statement

All animal experiments were approved by the University of Rochester Committee on Animal Resources (UCAR).

### 1. Rolling Repeat Algorithm for the Analysis of Mitochondrial Repeats

We take two copies of the mitochondrial sequence, either exactly the same (for DRs) or reverse complementary (for IRs), and denote as seq_1_ and seq_2_. First align them together, and then rotate them relative to each other one bp by one bp. For each rotation state, we do local alignments throughout the sequences and count all matched sequences. So, if two genomic positions are repeats, then there will be two rotation states for DRs in which one repeat sequence on seq_1_ will overlap with the other repeat on seq_2_, and one rotation state for IRs in which the two repeat sequences on seq_1_ will simultaneously overlap those on seq_2_ (Fig. S3). So, after a full rotation circle, each possible repeat will be counted exactly twice. The main algorithm in pseudocode is in Text S1.

For the local alignment, we reward a score of 1 for a match, and punish a score of 3 for a mismatch as the default condition as done by standard BLAST search. Although RollingRepeat allows extending the repeats in a low match region for 20 bps as long as the sequence at the end has high match quality, the stringency is not too low. For example, long repeats in human mitochondrial genome identified by RollingRepeat have at most 3 mismatches, with the longest repeat being 18 matches in 21 bps. A repeat has to have at least 16 bps to have 3 mismatches.

Since mitochondrial genomes of some species have highly repetitive sequences, usually tandem repeats (DRs), in the D-loop regions, which sometimes were also not well sequenced, we counted repeats only between gene regions (marked with “gene”, “tRNA”, “rRNA” or “CDS” in their GenBank files) when calculating the mutagenic scores, so the D-loop was excluded when calculating the correlations. However, for the repeat maps, we counted repeats between all regions.

### 2. Self-BLAST Algorithm for Repeat Discovery

We used NCBI blastn program to do local BLAST. The program was configured for short repeat discovery. Since blastn gives very few hits when one takes the whole mitochondrial genome to BLAST into itself, we first cut the mitochondrial genome into 100 bp blocks without overlaps and then BLAST each of these blocks onto the whole mitochondrial genome. Then, we counted all hits in gene regions except for self-hits and sorted them into direct and inverted repeats. The exact command we used for blastn was as follows:

blastn -task ‘blastn-short’ -num_descriptions 500000000 -num_alignments 500000000 -ungapped -query query.fa -db blastdb -word_size 4 -evalue 1e300

### 3. Combining Different Lengths of Repeats into a Single Mutagenic Score

We used a power function *l^n^* to fit published data about the relationship between direct repeat lengths *l* and deletion rate in yeast mitochondria (Fig. S4). We found that deletion rate is roughly proportional to *l* ^6^. Since *l* ^6^ can be an arbitrarily large number and the longest repeats (allowing only a few mismatches) in mammals are about 25 bps, (*l/*25)^6^ can be used to calculate the mutagenic score of each repeat so that the longest repeats will have a mutagenic score close to 1. Using this constant 25 will give us a sense of the total mutagenic potential a mitochondrial genome has, and will not change the correlation between total mutagenic score and lifespan. To allow a few mismatches, we used 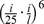 to calculate the mutagenic score of each repeat with *i* identical matches in a repeat of *l* bps, and *i/l* was used so that more mismatches would cause a reduced score (*i = l* if there were no mismatches). Then, the total mutagenic scores of DRs and IRs of a mitochondrial genome could be calculated, respectively.

### 4. Amplification and Sequencing of Inversions

We extracted total DNA from mouse brain, heart and liver tissues using DNeasy Tissue Kit (QIAGEN) with standard protocol. After PCR amplification with parallel primers (placed on the same mtDNA strand, synthesized by Integrated DNA Technologies), products were run on agarose gel. Different bands were cut and extracted using QIAEX II Gel Extraction Kit (QIAGEN). DNA extracted was directly sent for sequencing, or was cloned into plasmid using TOPO TA Cloning Kit (Invitrogen) and then sent for sequencing. For some primer pairs with a single pure amplified band, DNA was directly extracted from PCR product using QIAprep Spin Miniprep Kit (QIAGEN) and then cloned or directly sent for sequencing.

We originally designed primers between 22 to 30 bps and run PCR using an annealing temperature of about 50°C. Some inversions in Table 2 were amplified and sequenced by these primers. However, these primer pairs also gave nonspecific amplifications. Using longer primers (above 50 bps, usually below 60 bps) and a higher annealing temperature (above 60°C and below 72°C) allowed us to avoid nonspecific amplifications and primer dimer formation. Primers ended with G/C and were checked by program Amplify 3.1 (http://engels.genetics.wisc.edu/amplify/) on Mac OS X to avoid nonspecific amplifications of mtDNA and to reduce the chance of primer dimer formation, with the “Strigency” (stringency) on the “Dimers” tab set close to lowest to design primers with lowest dimer formation potential. Inversions were amplified using 45 thermal cycles of 92°C 20 s, 64°C 30 s and 72°C 1 min using parallel long primers and Taq DNA polymerase.

Long range (LR) PCR was performed using Expand Long Template PCR System (Roche). The two primers of LR single primer PCR used to amplify the last three products of Table 2 were 5′-2590CACCTTACAAATAAGCGCTCTCAACTTAATTTATGAATAAAATCTAAATAAAATATATACGTACACCCTCTAACC2664-3′ and 5′-5405CGCTCAGGCTCCGAATAGTAGATAGAGGGTTCCGATATCTTTGTGATTGGTTGAG5351-3′, respectively, with the first primer on the plus strand of mouse mitochondrial genome and the second primer on the minus strand. Brain total DNA of 30- or 24-month-old mice was used. The template was 0.2 µg of total genomic DNA. PCR was performed using the following thermal cycle: 92°C 2 min; 92°C 20 s, 68°C 4 min for 40 cycles; 68°C 5 min. PCR products were cloned into TA cloning vector for sequencing.

### 5. Quantification of Inversions using Regular PCR

Since primer dimers formed when inversion quantity was very low (as in young mice or liver), we combined regular PCR with agarose gel electrophoresis to compare inversions in different tissues or of different ages, instead of using real-time quantitative PCR. The PCR was performed using HotStarTaq Master Mix Kit (QIAGEN).

Equal amounts of total mtDNA were used as templates. The equal concentration of template DNA was verified using PCR with two primers that amplify a region of the mitochondrial genome. The reaction mix (20 µl) consisted of 10 µl Master Mix (2x), 0.1 µl of each primer (100 µM) and 9 µl ddH_2_O (double distilled water) and the adjusted volumes of templates (averaging 1 µl). The thermal cycle was: 94°C 10 min; 92°C 15 s, 65°C 15 s, 72°C 30 s for >40 cycles; 72°C 5 min.

Primer pairs used in Figure 3 were: Inversion 1, 5′-2590∼2664-3′ and 5′-5108∼5167-3′ (numbers indicate positions on mouse mitochondrial genome); Inversion 2, 5′-6791∼6842-3′ and 5′-8394∼8448-3′; Inversion 3, 5′-10680∼10626-3′ and 5′-12031∼11977-3′; Total mtDNA, 5′-12765∼12819-3′ and 5′-13127∼13073-3′.

### 6. Quantitative PCR (qPCR) Analysis

For this analysis we chose the inversion with the IR between positions 2742 and 5285 (Inversion 1). qPCR was done using GoTaq®qPCR Master Mix. The primer pair used to amplify this inversion was p_q1_ (5′-2604 GCGCTCTCAACTTAATTTATGAATAAAATCTAAATAAAATATATACGTACACCCTCTAACC2664-3′) and p_q2_ (5′-5203 GAGATTTCTCTACACCTTCGAATTTGCAATTCGACATGAATATCACCTTAAGACC5257-3′) as shown in Fig. 2e. Primer dimers did not form with this primer pair even after >40 thermal cycles.

The reaction mix (30 µl) consisted of 15 µl GoTaq® qPCR Master Mix, 0.15 µl CXR (passive reference dye), 0.088 µl each primer (100 µM), 14.2 µl ddH_2_O and 1∼2 µl template. The thermal cycle was: 95°C 5 min; 92°C 10 s, 70°C 30 s for 50 cycles, using 7300 Real-Time PCR System from Applied Biosystems.

To quantify the relative concentration of inversion #1 in total mtDNA in 30-month-old mouse brain, we made a 4X serial dilution of template to quantify total mtDNA. A 20X dilution of template was made to measure total DNA. The Ct values of serial dilutions and the 20X dilution were measured by the same qPCR system on the same plate. For inversion #1, a 4X serial dilution of plasmid containing inversion #1 was made, and the Ct values of serial dilutions and brain mtDNA were also measured by the same qPCR system on the same plate. We used the same threshold for both inversion and total mtDNA to calculate Ct values. Amplification efficiency of total mtDNA (*f_m_*) and inversion #1 (*f_i_*) were calculated from the serial dilutions according to Fig. S5a. Given a Ct value of the total mtDNA (*Ctm*) and inversion (*Cti*), the relative concentration of inversion #1 in mtDNA was calculated as *c* = *f_m_^Ctm^/*(20*f_i_^Cti^*).

### 7. Long Range (LR) PCR

The primers (100 µM) for LR PCR to amplify the inversion with IR between positions 2742 and 5285 were: p_LR1_, 5′-1167GTATTGGAGAAAGAAATTCGTACATCTAGGAGCTATAGAACTAGTACCGCAAGGGAAAG1225-3′; p_LR2_, 5′-6882CCTTCCTTTCTTATTTTACTTTTACATAGGTTGGTTCCTCGAATGTGTGATATGGTG6826-3′.

For the LR PCR, a 10 µl reaction system contained 8.57 µl ddH_2_O, 0.171 µl dNTP, 0.857 µl Buffer 1, 0.129 µl Taq, 0.171 µl template and 0.107 µl each primer (or using 0.107 µl ddH_2_O to replace a primer). We ran the LR PCR with the following thermal cycle: 94°C 2 min; 94°C 20 s, 62°C 30 s, 68°C 10 min for 8 or 12 cycles; 68°C 5 min; 4°C hold.

### 8. Nested PCR of LR PCR and qPCR to Quantify Inversion caused by mtDNA Replication Error

Three groups of reactions were performed simultaneously: Group B, p_LR1_+p_LR2_; Group S, p_LR1_; Group N, no primers. If inversions were simple inversions caused by homologous recombination, then they could only be amplified by Group B. However, if inversions were inside dimeric mtDNA circle caused by replication, then Group B and S would have the same efficiency in amplifying the inversion. p_LR2_ was 1.6 kb from the nearest primer in qPCR, so it would not interact with it in the 30 s annealing/extension time.

To ensure that B & S had the same concentration of p_LR1_ and that all three groups had the same concentration of template DNA, we first thoroughly mixed 300 µl ddH_2_O, 6 µl dNTP, 30 µl Buffer 1, 4.5 µl Taq and 6 µl template, and then took 65 µl of the mix out for Group N. Then we added 1.4 µl p_LR1_, mixed thoroughly, and took 130 µl out for Group B and S, respectively. Then we added 0.7 µl p_LR2_ to B, and 0.7 µl ddH_2_O to S and N, respectively. B and S were aliquoted into 12 PCR tubes and N into 6 PCR tubes, respectively, with 10 µl in each tube.

For the qPCR, we first prepared a master mix (28 µl for each reaction) without template for 86 reactions. After the LR PCR finished, we added 2 µl of the LR PCR product into 27.5 µl qPCR mix to dilute the LR PCR mix and primers. Each reaction of B and S was repeated three times in qPCR and each reaction of N was repeated twice. We used the median Ct value of the replicates as the Ct of the product of each LR PCR reaction.

Ct(B) and Ct(S) were denoted as the random variables of the Ct values of Group B and S (see Methods), with their standard deviations being *s*_Ct(B)_ and *s*_Ct(S)_, and their mean values being *M*_Ct(B)_ and *M*_Ct(S)_, respectively. Then, assuming normal distribution, we calculated the standard deviation of ΔCt = *M*_Ct(S)_ – *M*_Ct(B)_ as *s*_ΔCt_ = (*s* ^2^_Ct(S)_/12 + *s* ^2^_Ct(B)_/12)^0.5^.

Then, the maximum value of ΔCt with 95% confidence was calculated as *m* = norminv(0.95, ΔCt, *s*_ΔCt_) in MATLAB, and the corresponding minimum proportion of inversions caused by mtDNA replication was calculated as *f* ^–*m*^, where *f* was the qPCR amplification efficiency 1.67 (Fig. S5). The average proportion was calculated as *f* ^–ΔCt^.

### 9. LR qPCR System

The LR qPCR system was the same as in the nested PCR, but with two additional dyes: Double-strand DNA dye, EvaGreen (from Biotium, similar to SYBR Green); and a reference dye CXR (from GoTaq® qPCR Master Mix Kit, Promega).

### 10. Animals

C57BL/6 mice were obtained from the NIA aged rodent collection.

### 11. Statistics

The *p*-values of Pearson’s correlations were calculated by function [r, p] = corr(x, y), and the *p*-values of Spearman’s *ρ* was calculated by [r, p] = corr(x, y, ‘type’, ‘Spearman’) in MATLAB. The Shapiro-Wilk normality test of the residues was performed by shapiro.test in R. Our data usually did not meet normality assumption even after log transformation, but all data were log-transformed to improve normality. Comparative analysis by independent contrasts was performed using CAIC algorithm [31] by treating MLS as independent variable, and then Spearman’s *ρ* and *p*-values of the contrasts were calculated.

## Supporting Information

Figure S1 **IRs can be rotated to** **simultaneously match their reverse complementary sequences.** O, origin of the circular DNA.
(EPS)

Figure S2 **The correlations of DRs and IRs with MLS in all available animals. a**, the correlation for DRs. **b**, the correlation for IRs. Arthropods (all labeled) have high level of repeats, possibly because of their low body temperature and metabolic rates, although available data is not enough to be conclusive. *Caenorhabditis elegans* has a relatively high level of repeats, since its mitochondrial genome is much shorter than other species, with only about 13.8 k bps in total.
(EPS)

Figure S3 **Results generated by self-BLAST.** **a**, **b**, the correlation of DRs and IRs with MLS, respectively. **c**, the correlation of repeats with different lengths with MLS. **d**, **e**, repeat maps of both DRs (orange) and IRs (red) for mouse (*Mus musculus*) and human (*Homo sapiens*). Genomic annotations were extracted from the GenBank file of the reference genomes of the species in NCBI. Line widths are linear to the repeat lengths. Some repeats may be ignored because of the cutting process and because blastn does not output most short repeats (such as 1 ∼ 6 bp repeats). All repeats are a subset of repeats discovered by RollingRepeat even if we allowed extending a low match region of only 5 bps instead of the default 20 in RollingRepeat, confirming the robustness of the RollingRepeat algorithm.
(EPS)

Figure S4 **Empirical relationship between DR length and deletion rate in yeast mitochondria.** Since most animal repeats are short repeats and there are large errors for the longest repeats, we weighted more for short repeats in the curve fitting using MATLAB.
(EPS)

Figure S5 **Supplementary qPCR results.** **a**, qPCR Ct values of serial dilutions of the inversion amplified by primer pair of 5′-2604∼2664-3′ and 5′-5203∼5257-3′ (positions were on mouse mitochondrial genome) purified by QIAprep Spin Miniprep Kit. The slope was 1.35, so a 2-times increase in the replicon concentration needed 1.35 cycles. So, the amplification efficiency *f* of each cycle of the qPCR was 2^ 1*/*1.35^ = 1.67 (since *f* ^1.35^ = 2). **b**, agarose gel of LR qPCR products of group B and S. Arrays indicate the sizes of marker bands surrounding the expected bands. **c**, LR qPCR with template being 4-times serial dilutions of the purified DNA between 5 kb to 6 kb of LR PCR group B. No template controls were below the threshold (green horizontal arrow). Delta Rn, signal strength of double stranded DNA during annealing stage.
(EPS)

Text S1 (DOC)

## References

[1] Lee HC, Wei YH (2007) Oxidative stress, mitochondrial DNA mutation, and apoptosis in aging. Experimental biology and medicine 232: 592–606. 17463155

[2] Mammucari C, Rizzuto R (2010) Signaling pathways in mitochondrial dysfunction and aging. Mechanisms of Ageing and Development 131: 536–543. 20655326 10.1016/j.mad.2010.07.003 PMC2948971

[3] Harman D (1972) The biologic clock: the mitochondria? Journal of the American Geriatrics Society 20: 145–147. 5016631 10.1111/j.1532-5415.1972.tb00787.x

[4] Kujoth GC, Bradshaw PC, Haroon S, Prolla TA (2007) The role of mitochondrial DNA mutations in mammalian aging. PLoS genetics 3: e24. 17319745 10.1371/journal.pgen.0030024 PMC1802824

[5] Speakman JR, Selman C (2011) The free-radical damage theory: Accumulating evidence against a simple link of oxidative stress to ageing and lifespan. BioEssays : news and reviews in molecular, cellular and developmental biology 33: 255–259. 10.1002/bies.201000132 21290398

[6] Jang YC, Remmen VH (2009) The mitochondrial theory of aging: insight from transgenic and knockout mouse models. Experimental gerontology 44: 256–260. 19171187 10.1016/j.exger.2008.12.006

[7] Perez VI, Bokov A, Van Remmen H, Mele J, Ran QT, et al (2009) Is the oxidative stress theory of aging dead? Biochimica Et Biophysica Acta-General Subjects 1790: 1005–1014. 10.1016/j.bbagen.2009.06.003 PMC2789432 19524016

[8] Khrapko K, Vijg J (2009) Mitochondrial DNA mutations and aging: devils in the details? Trends Genet 25: 91–98. 19110336 10.1016/j.tig.2008.11.007 PMC2811092

[9] Khrapko K, Vijg J (2007) Mitochondrial DNA mutations and aging: a case closed? Nat Genet 39: 445–446. 17392805 10.1038/ng0407-445

[10] Khrapko K, Kraytsberg Y, de Grey AD, Vijg J, Schon EA (2006) Does premature aging of the mtDNA mutator mouse prove that mtDNA mutations are involved in natural aging? Aging Cell 5: 279–282. 16842501 10.1111/j.1474-9726.2006.00209.x

[11] Park CB, Larsson NG (2011) Mitochondrial DNA mutations in disease and aging. J Cell Biol 193: 809–818. 21606204 10.1083/jcb.201010024 PMC3105550

[12] Ameur A, Stewart JB, Freyer C, Hagstrom E, Ingman M, et al (2011) Ultra-deep sequencing of mouse mitochondrial DNA: mutational patterns and their origins. PLoS Genet 7: e1002028. 21455489 10.1371/journal.pgen.1002028 PMC3063763

[13] Dai DF, Santana LF, Vermulst M, Tomazela DM, Emond MJ, et al (2009) Overexpression of catalase targeted to mitochondria attenuates murine cardiac aging. Circulation 119: 2789–2797. 19451351 10.1161/CIRCULATIONAHA.108.822403 PMC2858759

[14] Osiewacz HD, Hamann A, Zintel S (2013) Assessing organismal aging in the filamentous fungus Podospora anserina. Methods Mol Biol 965: 439–462. 23296676 10.1007/978-1-62703-239-1_29

[15] Kuck U, Osiewacz HD, Schmidt U, Kappelhoff B, Schulte E, et al (1985) The onset of senescence is affected by DNA rearrangements of a discontinuous mitochondrial gene in Podospora anserina. Curr Genet 9: 373–382. 2836091 10.1007/BF00421608

[16] Osiewacz HD (2011) Mitochondrial quality control in aging and lifespan control of the fungal aging model Podospora anserina. Biochem Soc Trans 39: 1488–1492. 21936839 10.1042/BST0391488

[17] Kovalenko SA, Kopsidas G, Kelso JM, Linnane AW (1997) Deltoid human muscle mtDNA is extensively rearranged in old age subjects. Biochem Biophys Res Commun 232: 147–152. 9125120 10.1006/bbrc.1997.6251

[18] Linnane AW, Baumer A, Maxwell RJ, Preston H, Zhang CF, et al (1990) Mitochondrial gene mutation: the ageing process and degenerative diseases. Biochemistry international 22: 1067–1076. 1965280

[19] Vermulst M, Bielas JH, Kujoth GC, Ladiges WC, Rabinovitch PS, et al (2007) Mitochondrial point mutations do not limit the natural lifespan of mice. Nat Genet 39: 540–543. 17334366 10.1038/ng1988

[20] Trifunovic A, Wredenberg A, Falkenberg M, Spelbrink JN, Rovio AT, et al (2004) Premature ageing in mice expressing defective mitochondrial DNA polymerase. Nature 429: 417–423. 15164064 10.1038/nature02517

[21] Khrapko K, Bodyak N, Thilly WG, van Orsouw NJ, Zhang X, et al (1999) Cell-by-cell scanning of whole mitochondrial genomes in aged human heart reveals a significant fraction of myocytes with clonally expanded deletions. Nucleic Acids Res 27: 2434–2441. 10325435 10.1093/nar/27.11.2434 PMC148812

[22] Kraytsberg Y, Kudryavtseva E, McKee AC, Geula C, Kowall NW, et al (2006) Mitochondrial DNA deletions are abundant and cause functional impairment in aged human substantia nigra neurons. Nat Genet 38: 518–520. 16604072 10.1038/ng1778

[23] Vermulst M, Wanagat J, Kujoth GC, Bielas JH, Rabinovitch PS, et al (2008) DNA deletions and clonal mutations drive premature aging in mitochondrial mutator mice. Nat Genet 40: 392–394. 18311139 10.1038/ng.95

[24] Madsen CS, Ghivizzani SC, Hauswirth WW (1993) In vivo and in vitro evidence for slipped mispairing in mammalian mitochondria. Proc Natl Acad Sci U S A 90: 7671–7675. 8356068 10.1073/pnas.90.16.7671 PMC47204

[25] Larsson NG (2010) Somatic mitochondrial DNA mutations in mammalian aging. Annu Rev Biochem 79: 683–706. 20350166 10.1146/annurev-biochem-060408-093701

[26] Samuels DC (2004) Mitochondrial DNA repeats constrain the life span of mammals. Trends in genetics : TIG 20: 226–229. 15109774 10.1016/j.tig.2004.03.003

[27] Khaidakov M, Siegel ER, Reis RJS (2006) Direct repeats in mitochondrial DNA and mammalian lifespan. Mechanisms of Ageing and Development 127: 808–812. 16956646 10.1016/j.mad.2006.07.008

[28] de Magalhaes JP, Budovsky A, Lehmann G, Costa J, Li Y, et al (2009) The Human Ageing Genomic Resources: online databases and tools for biogerontologists. Aging Cell 8: 65–72. 18986374 10.1111/j.1474-9726.2008.00442.x PMC2635494

[29] Phadnis N, Sia RA, Sia EA (2005) Analysis of repeat-mediated deletions in the mitochondrial genome of Saccharomyces cerevisiae. Genetics 171: 1549–1559. 16157666 10.1534/genetics.105.047092 PMC1456083

[30] MITOMAP website: A Human Mitochondrial Genome Database. Available: http://www.mitomap.org. Accesses 2013 Mar 1.

[31] Purvis A, Rambaut A (1995) Comparative-Analysis by Independent Contrasts (Caic) - an Apple-Macintosh Application for Analyzing Comparative Data. Computer Applications in the Biosciences 11: 247–251. 7583692 10.1093/bioinformatics/11.3.247

[32] Thomas CAJ (1966) Recombination of DNA molecules. Prog Nucleic Acid Res Mol Biol 5: 315–337. 5337698 10.1016/s0079-6603(08)60237-8

[33] Lobachev KS, Shor BM, Tran HT, Taylor W, Keen JD, et al (1998) Factors affecting inverted repeat stimulation of recombination and deletion in Saccharomyces cerevisiae. Genetics 148: 1507–1524. 9560370 10.1093/genetics/148.4.1507 PMC1460095

[34] Schofield MA, Agbunag R, Miller JH (1992) DNA Inversions between Short Inverted Repeats in Escherichia-Coli. Genetics 132: 295–302. 1427029 10.1093/genetics/132.2.295 PMC1205136

[35] Williams SL, Huang J, Edwards YJ, Ulloa RH, Dillon LM, et al (2010) The mtDNA mutation spectrum of the progeroid Polg mutator mouse includes abundant control region multimers. Cell Metab 12: 675–682. 21109200 10.1016/j.cmet.2010.11.012 PMC3175596

[36] Bi X, Liu LF (1996) DNA rearrangement mediated by inverted repeats. Proceedings of the National Academy of Sciences of the United States of America 93: 819–823. 8570641 10.1073/pnas.93.2.819 PMC40140

[37] Bodyak ND, Nekhaeva E, Wei JY, Khrapko K (2001) Quantification and sequencing of somatic deleted mtDNA in single cells: evidence for partially duplicated mtDNA in aged human tissues. Hum Mol Genet 10: 17–24. 11136709 10.1093/hmg/10.1.17

