# Supplementary material for: Mitochondrial Inverted Repeats Strongly Correlate with Lifespan: mtDNA Inversions and Aging
Source: PLoS One. 2013 Sep 17;8(9):e73318. doi: 10.1371/journal.pone.0073318 (PMC3775743; doi:10.1371/journal.pone.0073318)
Supplement: Text S1 — (DOC) [file pone.0073318.s006.doc]

## Text S1. RollingRepeat algorithm for repeat discovery

Denote the genome size as *N* bps.

for (*n* = 1 to *N –* 1) do // *n*: number of bases rotated

*p* 🡨 1; // Genomic position

while (*p* ≤ *N*) // Find all repeats of this rotation state

while (seq_1_[*p*] ≠ seq_2_[*p*+*n*]) *p* 🡨 *p* + 1; // Find the first

// match of a repeat

*p*_0_ 🡨 *p*; // Save the start position of the repeat

continueExtending 🡨 TRUE;

score 🡨 0; // Local alignment score

maxS 🡨 0; // max score of this repeat when extending

*p_m_* = *p*; // End position of alignment with max score

while (continueExtending == TRUE)

if (seq_1_[*p*] ≠ seq_2_[(*p*+*n*)%*N*])

// %: Remainder of division

score 🡨 score + scoreOfAMatch;

else

score 🡨 score *–* punishmentOfAMismatch;

if (score > maxS) maxS = score, *p_m_* = *p*;

// Save the best alignment information

if (*p*>*p_m_*+20 OR *p>p_m_*+2(*p_m_*–*p*_0_+1) OR *p* ≥ *N*)

// Avoid long mismatches at the beginning

continueExtending 🡨 FALSE;

*p* 🡨 *p* + 1;

endwhile

Output information of this repeat (from *p*_0_ to *p_m_*);

*p* 🡨 *p_m_* + 1; // Prepare for the next repeat

endwhile

endfor

**Control experiments**

We ran six wells of the final qPCR products of each group on a gel; all the 18 wells had pure bands of the same expected size. We also purified DNA of two wells of each group and the sequencing confirmed that all the products were the same expected inversion. This ruled out nonspecific amplifications.

It was possible that Group B and Group S had similar Ct values simply because primer p_LR2_ did not work so that Group B and S had the same working primer (p_LR1_). To rule out this possibility, we did a LR qPCR with primer Group B and S. The template total DNA had a 4-time serial dilution. The well of Group B without dilution had a small Ct value of 12 (un-rearranged mtDNA could also be amplified by primer Group B, see Fig. 4), while the corresponding well of Group S had a Ct value of 40. This large difference of the Ct values suggests that primer p_LR2_ was efficiently amplifying the template. We ran the LR qPCR product on a gel; the first two wells of Group B had a pure band of the expected size (5.71 kb for B and 5.68 kb for S, Supplementary Figure S5b).

To further confirm the high amplification efficiency of primer p_LR2_ during the first 12 LR PCR cycles in Fig. 4b & 4c, we then purified DNA of the band with the expected size of group B and did a 4-times serial dilution. Further LR qPCR showed that the first few LR PCR had very high amplification efficiency (Figure S5c). Ct values of adjacent dilutions differed less than 2 cycles. This confirmed that primer p_LR2_ had high amplification efficiency during the first few LR PCR cycles, and the difference between the Ct values of Group B and S in Fig. 4 could only be explained by inversions being mainly caused by mtDNA replication error.
